# Combined predictive value of MRI parameters and inflammatory biomarkers in assessing the severity and prognosis of acute cervical traumatic

**DOI:** 10.1186/s12891-026-10362-6

**Published:** 2026-08-14

**Authors:** Yihao Liu, Xiaojuan Luo, Jinggang Le, Zhihui Zhong, Cong Xu, Daquan Zhou, Feng Chen

**Affiliations:** 1 Department of Neurosurgery, Xiangyang Central Hospital, Affiliated Hospital of Hubei University of Arts and Science, Xiangyang, China; 2 Department of Geriatrics, Xiangyang Central Hospital, Affiliated Hospital of Hubei University of Arts and Science, Xiangyang, China; 3 Department of Neurosurgery, the 1 st affiliated hospital, Jiangxi Medical College, Nanchang University, 17 Yongwai Street, Nanchang, Jiangxi 330006 China

**Keywords:** Cervical traumatic spinal cord injury, MRI variables, Inflammatory biomarkers

## Abstract

**Objective:**

This study evaluated the combined predictive value of preoperative MRI variables and preoperative peripheral inflammatory biomarkers in assessing the severity and prognosis of acute cervical TSCI.

**Methods:**

MRI parameters and peripheral inflammatory biomarkers were analyzed to determine their correlation with initial injury severity and 1-year follow-up prognosis. The predictive performance of different combinations of MRI variables and biomarkers was assessed using ROC analysis.

**Results:**

A total of 232 patients with acute cervical TSCI were included. MRI variables (including IMLL [intramedullary lesion length], BASIC [Brain and Spinal Cord Injury Center] score, MSCC [maximum spinal cord compression], MCC [maximum canal compromise], and sagittal grade system) and inflammatory biomarkers (including white blood cell [WBC] count, neutrophil count, platelet count, lymphocyte count, monocyte count, CRP [C-reactive protein], and novel inflammatory biomarkers (NLR[Neutrophil-to-lymphocyte ratio], PLR [Platelet-to-lymphocyte ratio], LMR [Lymphocyte-to-monocyte ratio] and SII [Systemic immune inflammatory index]) were significantly associated with initial injury severity. Univariate and multivariate logistic regression analyses identified BASIC score, severity of injury, SII, and NLR as independent predictors of poor prognosis. Notably, a model incorporating severity of injury, BASIC score, NLR, and SII demonstrated the highest predictive accuracy, yielding a superior area under the curve (AUC).

**Conclusions:**

The combination of the preoperative BASIC score, NLR, SII, and injury severity may serve as an exploratory preoperative prognostic model for neurological recovery after TSCI.

**Supplementary Information:**

The online version contains supplementary material available at https://doi.org/10.1186/s12891-026-10362-6.

## Introduction

Acute cervical traumatic spinal cord injury (TSCI) is a severe condition that frequently results in severe neurological deficits, diminished quality of life, and significant socioeconomic burdens [1, 2]. Accurate assessment of injury severity and reliable outcome prediction are crucial for optimizing treatment strategies and improving patient prognosis [3, 4]. However, the complex pathophysiological mechanisms underlying TSCI, including primary mechanical damage and secondary injury cascades such as inflammation, pose significant challenges to prognostic evaluation.

Magnetic resonance imaging (MRI) is widely employed for assessing acute TSCI, and numerous studies have explored the utility of MRI markers as neuroimaging biomarkers for clinical evaluation [5–10]. These studies have examined factors such as the length of high T2 signal within the spinal cord, whether the abnormal T2 signal is localized or extends across multiple vertebral levels, the presence of hemorrhage, and secondary indicators of TSCI, such as spinal canal and cord compression. These MRI parameters have demonstrated reproducibility and predictive validity for clinical prognosis. Therefore, integrating these imaging markers with clinical assessments can enhance decision-making in urgent treatment scenarios.

Simultaneously, systemic inflammation has emerged as a critical factor in TSCI pathophysiology. Following TSCI, a robust inflammatory response is triggered, characterized by peripheral immune cell activation and the release of inflammatory mediators [11]. Peripheral inflammation markers, including white blood cell (WBC) count, neutrophils, and C-reactive protein (CRP), are associated with injury severity and neurological recovery in patients with TSCI [12–14]. Novel inflammatory biomarkers, such as the neutrophil-to-lymphocyte ratio (NLR), platelet-to-lymphocyte ratio (PLR), lymphocyte-to-monocyte ratio (LMR), and systemic immune-inflammation index (SII), have been identified as indicators of systemic immune activation and prognostic markers. These biomarkers have been applied in evaluating disease severity and prognosis across various conditions, including neurological disorders [15, 16], tumors [17–19], cardiovascular diseases [20, 21], and respiratory diseases [22, 23].

While substantial evidence supports the prognostic value of MRI and inflammatory biomarkers individually, their combined application in predicting the severity and outcomes of acute cervical TSCI remains largely unexplored. Integrating these two modalities could enhance our understanding of TSCI pathophysiology and improve the accuracy of prognostic models. Examining the interplay between structural damage detected via MRI and systemic inflammatory responses may provide novel insights into the mechanisms underlying TSCI progression and recovery. This study aimed to assess the predictive value of MRI parameters and peripheral inflammatory biomarkers in determining the severity and prognosis of acute cervical TSCI. By integrating metrics from these two modalities, a more comprehensive framework was sought to be developed for early risk stratification and personalized treatment planning, ultimately enhancing prognostic accuracy in TSCI management.

## Materials and methods

### Study population

This study enrolled 232 patients with acute cervical TSCI admitted to the First Affiliated Hospital of Nanchang University between January 2018 and October 2023. The inclusion criteria were as follows:1) traumatic etiology; 2) a Glasgow Coma Scale (GCS) score of 15; 3) confirmed acute TSCI; and peripheral blood sample collection within 24 h and MRI examination within 48 h of injury. 5)All underwent surgical treatment.Exclusion criteria included: (1) age < 17 years; (2) severe multiple injuries requiring invasive interventions that precluded reliable AIS assessment; (3) pre-existing hematologic disorders; and (4) incomplete or missing data. The Ethics Committee of the First Affiliated Hospital of Nanchang University approved this study. Only preoperative MRI examinations obtained within 48 h after injury were used for MRI parameter assessment. Patients with missing essential clinical, laboratory, imaging, or follow-up data were excluded. No statistical imputation was performed, and all analyses were conducted using complete-case data.Peripheral venous blood samples were collected within 24 h after injury and before surgery. All procedures were conducted following relevant guidelines and regulations.Neurological assessment was performed using the American Spinal Injury Association Impairment Scale (AIS) following the International Standards for Neurological Classification of Spinal Cord Injury (ISNCSCI). AIS grades for all patients were independently evaluated by two neurosurgical specialists upon hospital admission.Because of the retrospective design, a standardized repeat AIS examination after the resolution of spinal shock was not consistently available for all patients. Therefore, the admission AIS grade was used for baseline severity classification.Based on prior research, cervical TSCI severity was categorized into two groups: severe TSCI (AIS grades A-B) and non-severe TSCI (AIS grades C-E) [5]. Clinical data collected included patient demographics (age, gender), injury mechanism, AIS grades at admission and follow-up, laboratory findings, and quantitative MRI parameters. Because of the retrospective design and incomplete standardization of clinical records during the study period, complete information regarding the interval from injury to decompression, duration of surgery, surgical approach, perioperative mean arterial pressure management, and corticosteroid administration was not available for all patients. These treatment-related variables were therefore not included in the multivariable analysis.The primary outcome measure was the AIS grade change one-year post-TSCI, aligning with the National Acute Spinal Cord Injury Study (NASCIS) guidelines. The 1-year neurological outcome was assessed through outpatient follow-up or structured telephone follow-up. During outpatient follow-up, neurological function was evaluated by direct motor and sensory examination. For patients who were unable to return for an in-person visit, structured telephone follow-up was conducted to assess upper- and lower-extremity motor and sensory function based on standardized questions.Patients with sufficient follow-up information were classified into two neurological outcome groups: favorable outcome (AIS grades D–E) and unfavorable outcome (AIS grades A–C) [24, 25]. This binary classification was used to reflect clinically meaningful favorable versus unfavorable neurological outcomes and to facilitate direct comparison between patients with good and poor prognosis.

### Clinical data collection

Peripheral blood samples were obtained from all patients with TSCI within 24 h after injury and before surgery. The laboratory tests analyzed included WBC, neutrophil, lymphocyte, monocyte, and platelet counts, and CRP. These tests were conducted in the Clinical Laboratory Department of the First Affiliated Hospital of Nanchang University. Novel inflammatory markers were calculated using the following formulas: NLR is calculated by dividing the neutrophil count by the lymphocyte count; PLR is found by dividing the platelet count by the lymphocyte count; LMR is calculated by dividing the lymphocyte count by the monocyte count; SII is derived by multiplying the platelet count by the neutrophil count and dividing the result by the lymphocyte count.

### MRI variables

**Fig. 1:**
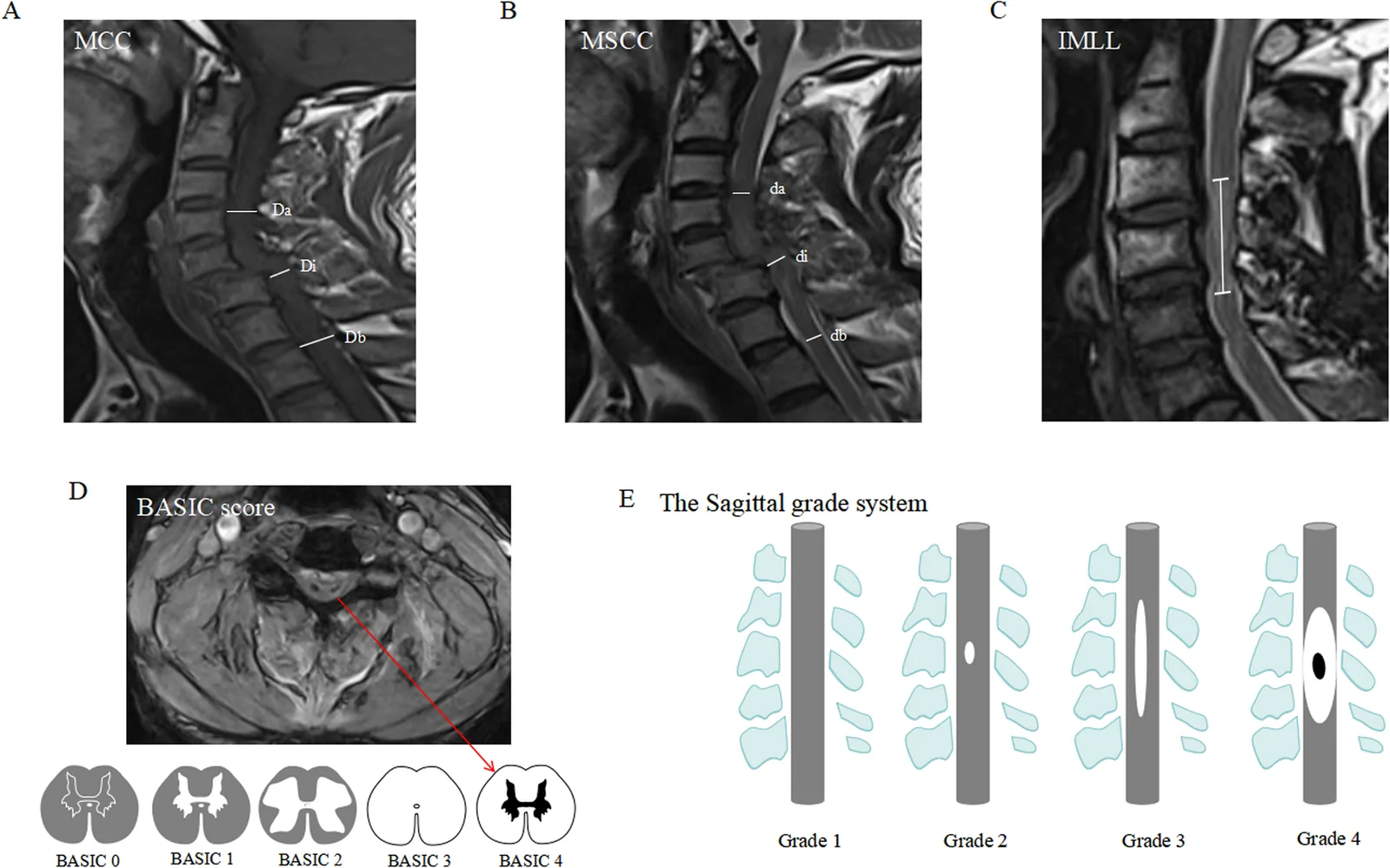
MRI parameter illustrations-(**A**) MCC (%) = 1 – (Di/[Da + Db]/2) × 100; (**B**) MSCC (%) = 1 – (di/[da + db]/2) × 100; (**C**) IMLL; (**D**) BASIC score; (**E**) Sagittal grade system. BASIC, Brain and Spinal Injury Center; IMLL, intramedullary lesion length; MRI, magnetic resonance imaging; MSCC, maximum spinal cord compression; MCC, maximum canal compromise

Five MRI parameters were analyzed as potential predictors of injury severity and prognosis: (1) maximum canal compromise (MCC), (2) maximum spinal cord compression (MSCC), (3) intramedullary lesion length (IMLL), (4) Brain and Spinal Injury Center (BASIC) score, and (5) sagittal grade. All MRI examinations used for these measurements were performed preoperatively. The MCC was calculated by dividing the anteroposterior diameter of the spinal canal (measured on sagittal T1-weighted images) by the average diameter of the spinal canal in the sagittal view (Fig. 1A). The MSCC was obtained by dividing the anteroposterior diameter of the spinal cord (measured on sagittal T2-weighted images) by the average diameter of the spinal cord above and below the lesion [10] (Fig. 1B). The following formulas were used: MCC (%) = 1 – (Di/[Da + Db]/2) × 100; MSCC (%) = 1 – (di/[da + db]/2) × 100 (Fig. 1). The IMLL refers to the longitudinal extent of the midline sagittal T2 signal alteration following acute TSCI, encompassing spinal cord edema, contusion, and hemorrhage [5] (Fig. 1A, C). In 2014, Talbott et al. [9] introduced the BASIC score, an axial plane grading system for assessing spinal cord injury severity. The BASIC score ranges from 0 to 4 based on T2 signal changes within the spinal cord (Grade 1: signal change confined to the central grey matter; Grade 2: signal extends beyond the grey matter into the white matter but does not involve the entire spinal cord; Grade 3: signal alteration spans the entire transverse extent of the spinal cord; Grade 4: Grade 3 features with additional T2 low-signal foci indicative of hemorrhage (Fig. 1D). Similarly, Haefeli et al. proposed a sagittal plane grading system to categorize injury severity based on T2 intramedullary signal alterations: Grade 1: normal spinal cord signal; Grade 2: signal change limited to a single vertebral level; Grade 3: signal alteration extending beyond one vertebral level; Grade 4: presence of both edema and hemorrhage [10] (Fig. 1E). All AIS grades for all patients were independently evaluated by two neurosurgical specialists upon hospital admission. Disagreements were resolved through discussion and consensus.

### Statistical analysis

Categorical data were summarized as frequencies and percentages, while continuous variables were reported as mean ± standard deviation (SD) or median with interquartile range (IQR). The Shapiro–Wilk test was applied to assess data normality. For normally distributed variables, comparisons were conducted using Student’s t-test or analysis of variance (ANOVA). Conversely, non-normally distributed variables were analyzed using the Kruskal–Wallis or Mann–Whitney U tests. This study aimed to evaluate the correlation between MRI parameters, inflammatory biomarkers, and TSCI severity and prognosis. Spearman’s rank correlation was employed to assess bivariate correlations. Univariate analysis identified significant variables, and those with a p-value < 0.1 were incorporated into the multivariate logistic regression model. The predictive performance of different risk factor combinations for AIS classification at follow-up was assessed using receiver operating characteristic (ROC) curve analysis. All statistical analyses were conducted using SPSS software (version 21.0; IBM), with statistical significance set at a two-sided p-value < 0.05.To assess the internal validity and robustness of the predictive model, we performed bootstrap resampling with 1000 iterations. Model calibration was evaluated using the Hosmer-Lemeshow goodness-of-fit test and visualized via a calibration plot. Additionally, a nomogram was constructed to facilitate clinical application, and risk stratification was performed based on the optimal probability cut-off determined by the Youden index.

## Results

**Fig. 2:**
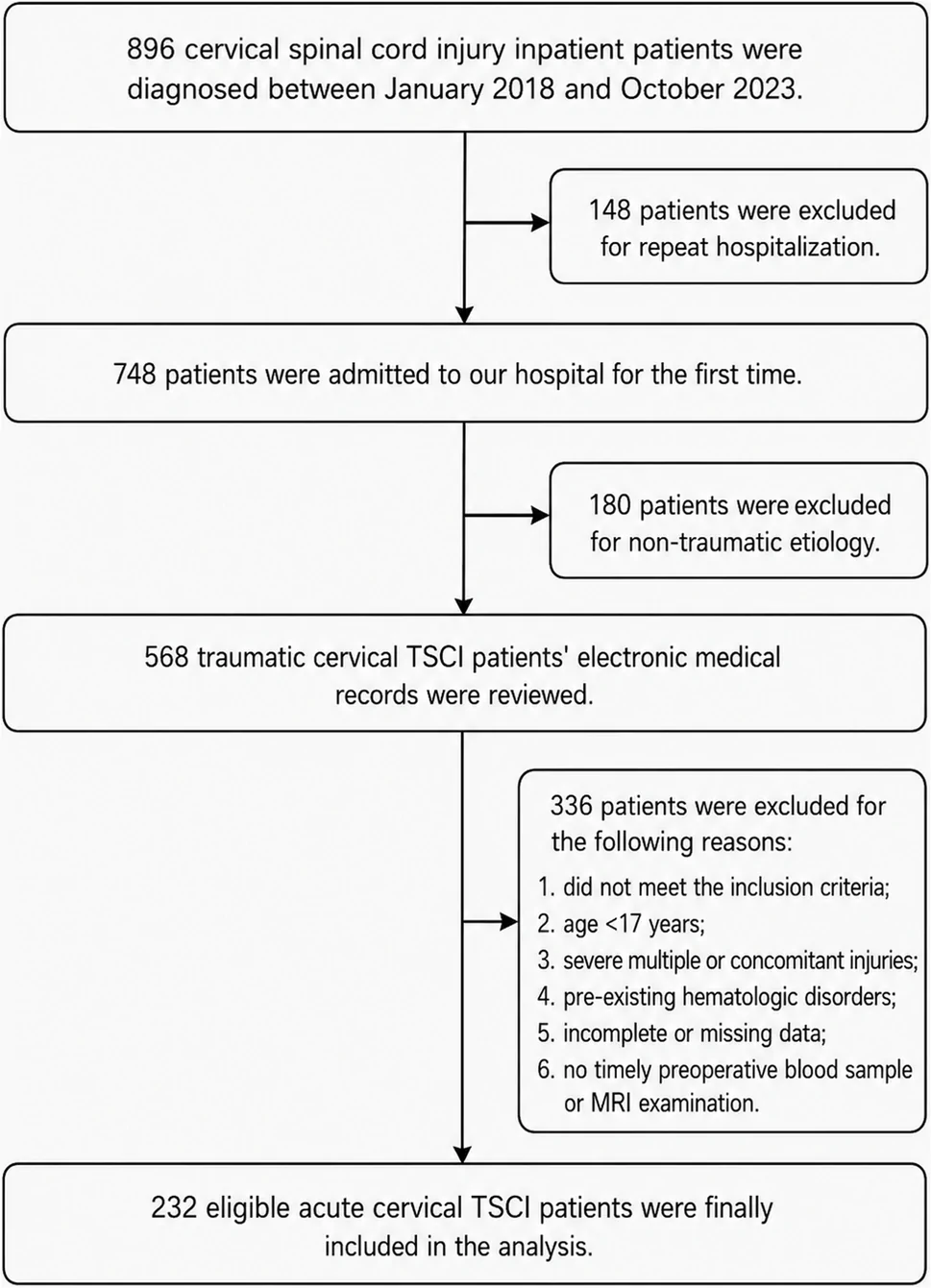
Patient selection flowchart

This study included 232 patients with acute cervical TSCI (Fig. 2), comprising 201 men and 31 women. Table 1 summarizes the patients’ essential characteristics, MRI parameters, and peripheral inflammatory biomarkers. The mean age was 54.67 years (17–84 years). The most common injury mechanism was falls (44.4%), followed by motor vehicle accidents (34.5%), object impacts (5.6%), and other unspecified causes (15.5%). At admission, the AIS grade distribution was as follows: AIS A in 90 patients (38.8%), AIS B in 33 (14.2%), AIS C in 37 (15.9%), and AIS D in 72 (31.1%). After 1-year, the distribution changed to AIS A in 60 patients (25.9%), AIS B in 12 (5.2%), AIS C in 27 (11.6%), AIS D in 113 (48.7%), and AIS E in 20 (8.6%). Table 1 details MRI parameters and inflammatory biomarkers. The mean values of IMLL, MCC, and MSCC were 24.29 mm (SD ± 22.18 mm), 34.51% (SD ± 22.62%), and 19.78% (SD ± 17.44%), respectively. The BASIC scores of 0, 1, 2, 3, and 4 were distributed across 40 (17.2%), 25 (10.8%), 52 (22.4%), 30 (12.9%), and 85 (36.6%), respectively. Sagittal grading showed Grades 1, 2, 3, and 4 in 40 (17.2%), 59 (25.4%), 48 (20.7%), and 85 (36.6%) patients, respectively. Regarding inflammatory biomarkers, the mean values for WBC, neutrophil, lymphocyte, monocyte, and platelet counts (all ×10⁹/L), and CRP (mg/L) were 10.57 (SD ± 3.15), 9.31 (SD ± 3.13), 0.85 (SD ± 0.53), 0.39 (SD ± 0.34), 187.99 (SD ± 61.97), and 15.81 (SD ± 25.24), respectively. Moreover, the novel inflammatory markers NLR, PLR, LMR, and SII had mean values of 14.91 (SD ± 10.03), 281.94 (SD ± 168.64), 4.54 (SD ± 6.24), and 2720.14 (SD ± 1944.36), respectively.Patients with severe concomitant injuries that could interfere with reliable AIS grading or imaging-based neurological assessment were excluded according to the predefined criteria.

**Table 1:** Patient characteristics

Mean age (range)(years)	54.44 ± 11.40 (17–84)
Sex (%)	Male = 201 (90.2); Female = 31 (9.8)
Mechanism of injury (%)	Fall = 103 (44.4); MVA = 80 (34.5); Struck-by-object = 13 (5.6); Others = 36 (15.5)
Admission AIS grade (%)	A = 90 (38.8); B = 33 (14.2); C = 37 (15.9); D = 72 (31.0)
1-year AIS grade (%)	A = 60 (25.9); B = 12 (5.2); C = 27 (11.6); D = 113 (48.7); E = 20 (8.6)
BASIC grade (%)	0 = 40 (17.2); 1 = 25 (10.8); 2 = 52 (22.4); 3 = 30 (12.9); 4 = 85 (36.6)
Sagittal grade (%)	1 = 40 (17.2); 2 = 59 (25.4); 3 = 48 (20.7); 4 = 85 (36.6)
surgery (%)	Yes = 232(100);No = 0 (0)
IMLL(mm) (mean (SD))	24.29 ± 22.18
MCC (%) (mean (SD))	34.51 ± 22.62
MSCC (%) (mean (SD))	19.78 ± 17.44
WBC (×10^9^/L) (mean (SD))	10.57 ± 3.15
Neutrophils (×10^9^/L) (mean (SD))	9.31 ± 3.13
Lymphocytes (×10^9^/L) (mean (SD))	0.85 ± 0.53
Monocytes(×10^9^/L) (mean (SD))	0.39 ± 0.34
Platelets(×10^9^/L) (mean (SD))	187.99 ± 61.97
CRP (mg/L) (mean (SD)	15.81 ± 25.24
NLR (mean (SD))	14.91 ± 10.03
PLR (mean (SD))	281.94 ± 168.64
LMR (mean (SD))	4.54 ± 6.24
SII (×10^9^/L) (mean (SD))	2720.14 ± 1944.36

*AIS* American spinal injury association Impairment Scale, *IMLL* Intramedullary lesion length, *LMR* Lymphocyte-to-monocyte ratio, *MCC* Maximum canal compromise, *MSCC* Maximum spinal cord compression, *NLR* Neutrophil-to-lymphocyte ratio, *PLR* Platelet-to-lymphocyte ratio, *SD* Standard deviation, *SII* Systemic immune inflammatory index, *WBCs* White blood cells

Based on our previous study [25], 123 patients (53%) were classified as severe TSCI (AIS A-B), and 109 (47%) as non-severe TSCI (AIS C-E) (Table 2). Significant differences were observed between these groups in admission AIS grade, MCC, IMLL, BASIC score, sagittal grade, WBC, neutrophil, lymphocyte, monocyte, and platelet counts, CRP, NLR, PLR, and SII (Table 2). To further investigate the relationship between MRI parameters, inflammatory biomarkers, and admission AIS grade, MRI variables (IMLL, BASIC score, Sagittal grade, and MCC) were found to decrease progressively as the AIS grade improved. Similarly, increasing AIS grade significantly declined novel inflammatory biomarkers (Table 3). Notably, the BASIC score demonstrated a stronger correlation with admission AIS grade than other MRI parameters, while NLR was the most strongly associated inflammatory biomarker (Table 3).

**Table 2:** Comparative analysis of clinical features and laboratory findings in severe versus non-severe cervical TSCI

Characteristics	Non-severe TSCI	Severe TSCI	*P* value
*n*	109	123	
Sex, *n* (%)			0.099
Males	89 (38.4%)	112 (48.3%)	
Females	20 (8.6%)	11 (4.7%)	
Age, median (± SD)	54.19 ± 11.81	54.65 ± 11.06	0.773
Mechanism of injury, *n* (%)			0.158
Fall	43 (18.5%)	60 (25.9%)	
Motor vehicle accident	44 (19%)	36 (15.5%)	
Struck-by-object	3 (1.3%)	10 (4.3%)	
Others	19 (8.2%)	17 (7.3%)	
IMLL, median (± SD)	12.20 ± 15.31	35.00 ± 21.85	< 0.001
MSCC(%), median (± SD)	17.58 ± 14.29	21.73 ± 19.69	0.130
MCC(%), median (± SD)	27.7 ± 17.54	40.53 ± 24.87	< 0.001
BASIC score			< 0.001
0	40 (17.24%)	0 (0%)	
1	22 (9.48%)	3 (1.29%)	
2	36 (15.52%)	16 (6.90%)	
3	6 (2.59%)	24 (10.34%)	
4	5 (2.16%)	80 (34.48%)	
Sagittal grade			< 0.001
1	40 (17.24%)	0 (0%)	
2	43 (18.53%)	16 (6.90%)	
3	21 (9.52%)	27 (11.64%)	
4	5 (2.16%)	80 (34.48%)	
WBC (×10^9^/L), median (± SD)	9.99 ± 3.11	11.09 ± 3.11	0.045
Neutrophils (×10^9^/L), median (± SD)	8.52 ± 3.18	10.02 ± 2.92	0.002
Lymphocytes (×10^9^/L), median (± SD)	1.02 ± 0.63	0.7 ± 0.35	< 0.001
Monocytes(×10^9^/L), median (± SD)	0.44 ± 0.35	0.35 ± 0.32	< 0.001
Platelets(×10^9^/L), median (± SD)	195.77 ± 63.52	181.09 ± 59.98	< 0.001
CRP (mg/L), median (± SD)	13.12 ± 23.43	18.19 ± 26.62	< 0.001
NLR, median (± SD)	12.34 ± 9.92	17.19 ± 9.61	< 0.001
PLR, median (± SD)	109 ± 281.94	305.00 ± 164.58	0.014
LMR, median (± SD)	4.52 ± 5.73	4.55 ± 6.68	0.380
SII, median (± SD)	2328.86 ± 1950.01	3066.89 ± 1879.91	0.002

*AIS* American spinal injury association Impairment Scale, *IQR* Interquartile ranges, *IMLL* Intramedullary lesion length, *LMR* Lymphocyte-to-monocyte ratio, *MCC* Maximum canal compromise, *MSCC* Maximum spinal cord compression, *SD* Standard deviation, *SII* Systemic immune inflammatory index, *WBCs* White blood cells

**Table 3:** Spearman rank correlation and regression analysis results

Admission AIS grade	Spearman correlation	
	*R*	*P*
IMLL	−0.589	< 0.001
BASIC	−0.827	< 0.001
MSCC	−0.032	0.625
MCC	−0.289	< 0.001
Sagittal grade	−0.778	< 0.001
WBCs	−0.180	0.06
Neutrophils	−0.247	0.001
Lymphocytes	0.327	< 0.001
Monocytes	0.232	< 0.001
Platelets	0.176	0.007
CRP	−0.185	0.005
NLR	−0.368	< 0.001
PLR	−0.240	0.004
LMR	−0.017	0.799
SII	−0.291	< 0.001
1-year AIS grade	Spearman correlation	
	R	*P*
IMLL	−0.586	< 0.001
BASIC	−0.797	< 0.001
MSCC	−0.067	0.307
MCC	−0.338	< 0.001
Sagittal grade	−0.779	< 0.001
WBCs	−0.160	0.015
Neutrophils	−0.227	0.001
Lymphocytes	0.328	< 0.001
Monocytes	0.236	< 0.001
Platelets	0.252	< 0.001
CRP	−0.258	< 0.001
NLR	−0.355	< 0.001
PLR	−0.187	0.004
LMR	−0.011	0.869
SII	−0.235	< 0.001

*IMLL* Intramedullary lesion length, *LMR* Lymphocyte-to-monocyte ratio, *MCC* Maximum canal compromise, *MSCC* Maximum spinal cord compression, *NLR* Neutrophil-to-lymphocyte ratio, *PLR* Platelet-to-lymphocyte ratio, *SII* Systemic immune inflammatory index, *WBCs* White blood cells

**Fig. 3:**
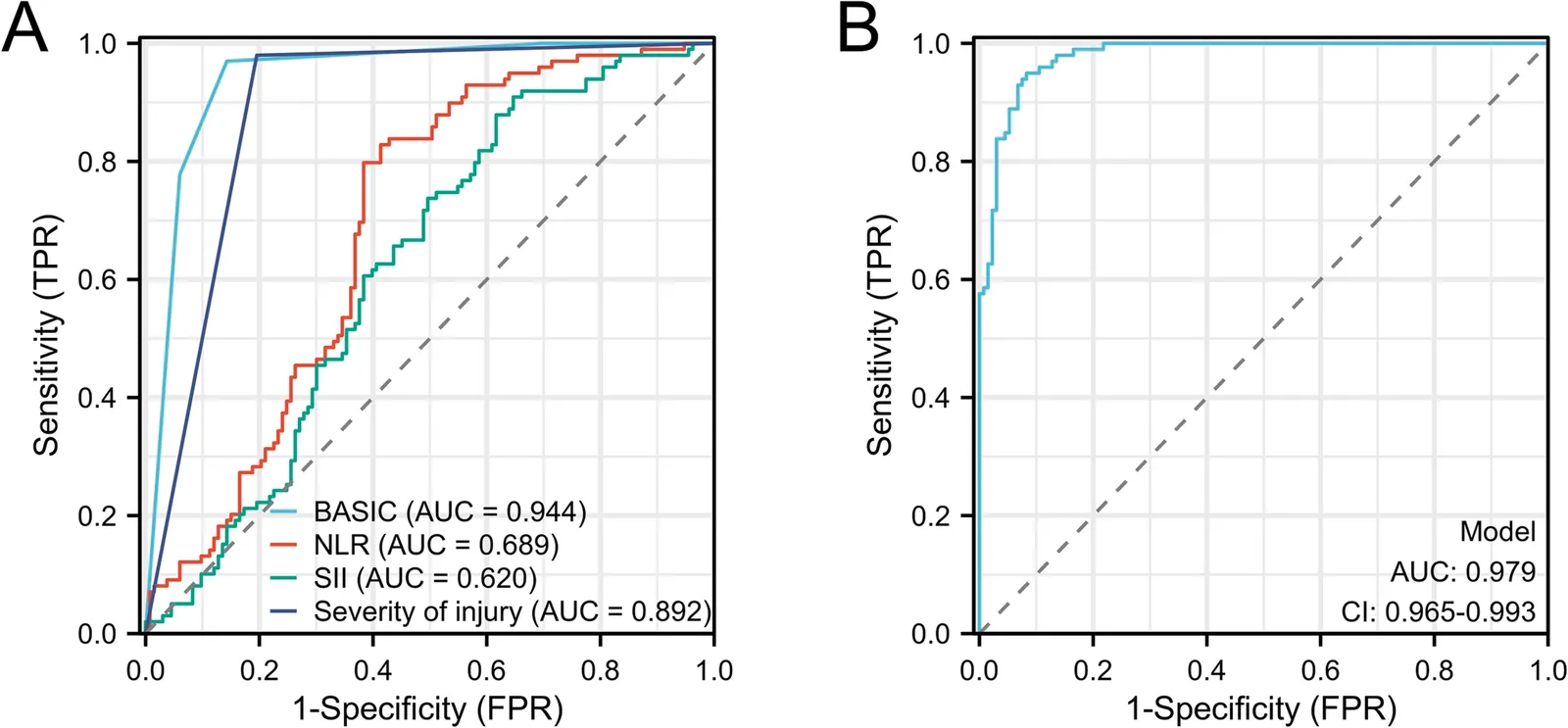
**A** The ROC curve for predicting TSCI prognosis using BASIC score, injury severity, NLR, and SII. **B** The combined model (severity of injury, BASIC score, NLR, and SII) achieves a larger AUC, indicating superior predictive performance. IMLL, Intramedullary lesion length; MRI, Magnetic resonance imaging; NLR, Neutrophil-to-lymphocyte ratio; ROC, receiver operating characteristic; MSCC, maximum spinal cord compression; MCC, maximum canal compromise; BASIC, Brain and Spinal Injury Center; NLR, neutrophil-to-lymphocyte ratio; SII, systemic immune-inflammation index; TSCI, traumatic spinal cord injury

At the 1-year follow-up, 99 patients (42.7%) had a poor prognosis (AIS A–C), while 133 (57.3%) had a favorable outcome (AIS D–E). Analysis of MRI variables and inflammatory biomarkers concerning the follow-up AIS grade revealed that worsening prognosis was associated with more severe MRI findings, with the BASIC score showing the strongest correlation among IMLL, MCC, and sagittal grade (Table 3). Regarding inflammatory biomarkers, WBC and neutrophil counts, CRP, NLR, PLR, and SII increased with worsening prognosis, whereas lymphocyte, monocyte, and platelet counts were higher in patients with better outcomes (Table 3). Univariate analysis identified substantial differences between patients with good and poor prognosis in injury severity, IMLL, MCC, BASIC score, sagittal grade, and inflammatory markers (Table 4). After adjusting for confounders, stepwise multivariate logistic regression identified injury severity, BASIC score, NLR, and SII as independent predictors of 1-year prognosis in patients with TSCI (Table 5). Furthermore, ROC analysis was performed to evaluate the discriminatory performance of the BASIC score, NLR, SII, admission injury severity, and their combined model for predicting the 1-year neurological outcome. As shown in (Figure 3A), the ROC values were ༈0.944༉ for the BASIC score, ༈0.689༉ for NLR, ༈0.620༉ for SII, and ༈0.892༉ for admission injury severity. Among the individual predictors, the BASIC score demonstrated the highest discriminatory performance, whereas NLR and SII showed relatively limited discrimination when used alone. The combined model incorporating admission injury severity, BASIC score, NLR, and SII achieved a ROC of ༈0.979༉ (95% CI, 0.965–0.993), indicating higher apparent discriminatory performance than any individual predictor (Fig. 3B).

**Table 4:** Results of statistical analysis of the risk factors for poor prognosis in cervical TSCI

Characteristics	Good outcome	Poor outcome	*P* value
*n*	133	99	
Sex, *n* (%)			0.099
Males	111 (47.8%)	90 (38.8%)	
Females	22 (9.5%)	9 (3.9%)	
Age, mean ± SD	54.25 ± 11.27	54.69 ± 11.62	0.773
Mechanism of injury, *n* (%)			0.158
Fall	51 (22%)	52 (22.4%)	
Motor vehicle accident	52 (22.4%)	28 (12.1%)	
Struck-by-object	7 (3%)	6 (2.6%)	
Others	23 (9.9%)	13 (5.6%)	
Severity of TSCI, *n* (%)			< 0.001
Non-severe cervical TSCI	107 (46.1%)	2 (0.9%)	
Severe cervical TSCI	26 (11.2%)	97 (41.8%)	
IMLL, mean ± SD	15.53 ± 16.30	38.72 ± 20.83	< 0.001
MSCC(%), mean ± SD	17.80 ± 15.10	22.44 ± 19.93	
MCC(%), mean ± SD	27.64 ± 19.52	43.73 ± 23.30	< 0.001
BASIC			< 0.001
0	40 (17.24%)	0 (0%)	
1	24 (10.34%)	1 (0.43%)	
2	50 (21.55%)	2 (0.86%)	
3	11 (4.74%)	19 (8.19%)	
4	8 (3.45%)	77 (33.19%)	
Sagittal grade, *n* (%)			< 0.001
1	40 (17.24%)	0 (0%)	
2	54 (23.28%)	5 (2.16%)	
3	31 (13.36%)	17 (7.33%)	
4	8 (3.45%)	77 (33.19%)	
WBC (×10^9^/L), mean ± SD)	10.40 ± 3.42	10.81 ± 2.76	0.045
Neutrophils (×10^9^/L, mean ± SD)	8.93 ± 3.42	9.83 ± 2.63	0.002
Lymphocytes (×10^9^/L), mean ± SD	0.99 ± 0.60	0.67 ± 0.40	< 0.001
Monocytes (×10^9^/L), mean ± SD	0.47 ± 0.38	0.29 ± 0.23	< 0.001
Platelets (×10^9^/L), mean SD	201.22 ± 65.80	170.21 ± 51.62	< 0.001
CRP (mg/L), mean ± SD	13.02 ± 22.61	19.55 ± 28.10	< 0.001
NLR, mean ± SD	12.75 ± 9.52	17.81 ± 10.02	< 0.001
PLR, mean ± SD	264.71 ± 166.31	305.10 ± 169.82	0.014
LMR, mean ± SD	4.61 ± 6.89	4.44 ± 5.27	0.380
SII (×10^9^/L), mean ± SD	2517.84 ± 2029.16	2991.92 ± 1798.65	0.002

*AIS* American spinal injury association Impairment Scale, *IQR* Interquartile ranges, *IMLL* Intramedullary lesion length, *LMR* Lymphocyte-to-monocyte ratio, *MCC* Maximum canal compromise, *MSCC* Maximum spinal cord compression, *SD* Standard deviation, *SII* Systemic immune inflammatory index, *WBCs* White blood cells

**Table 5:** Multivariate logistic regression analysis for risk factors of poor outcome in cervical TSCI

Characteristics	OR (95% CI)	*P* value
Sex	2.73 (0.26–28.87)	0.403
Severity of injury		< 0.001
IMLL	1.02 (0.96–1.09)	0.47
MSCC	1.01 (0.93–1.10)	0.81
MCC	0.98 (0.91–1.05)	0.587
BASIC Score		0.009
Sagittal grade		0.262
WBC	938.69 (0.01–183000567.2)	0.271
Neutrophils	0.001 (0.00–154.93)	0.252
Lymphocytes	0.00 (0.00–84.41)	0.191
Monocytes	1.85 (0.00–265150.07)	0.919
Platelets	0.96 (0.91–1.01)	0.075
CRP	0.10 (0.96–1.03)	0.830
NLR	0.52 (0.29–0.94)	0.029
PLR	0.99 (0.97–1.02)	0.515
LMR	1.18 (0.92–1.51)	0.188
SII	1.00 (1.00–1.01)	0.014

*AIS* American spinal injury association Impairment Scale, *BASIC* Brain and spinal cord injury center, *IMLL* Intramedullary lesion length, *ROC* Receiver operating characteristic, *MCC* Maximum canal compromise, *MSCC* Maximum spinal cord compression, *WBCs* White blood cells

### Model validation and clinical tools

**Fig. 4:**
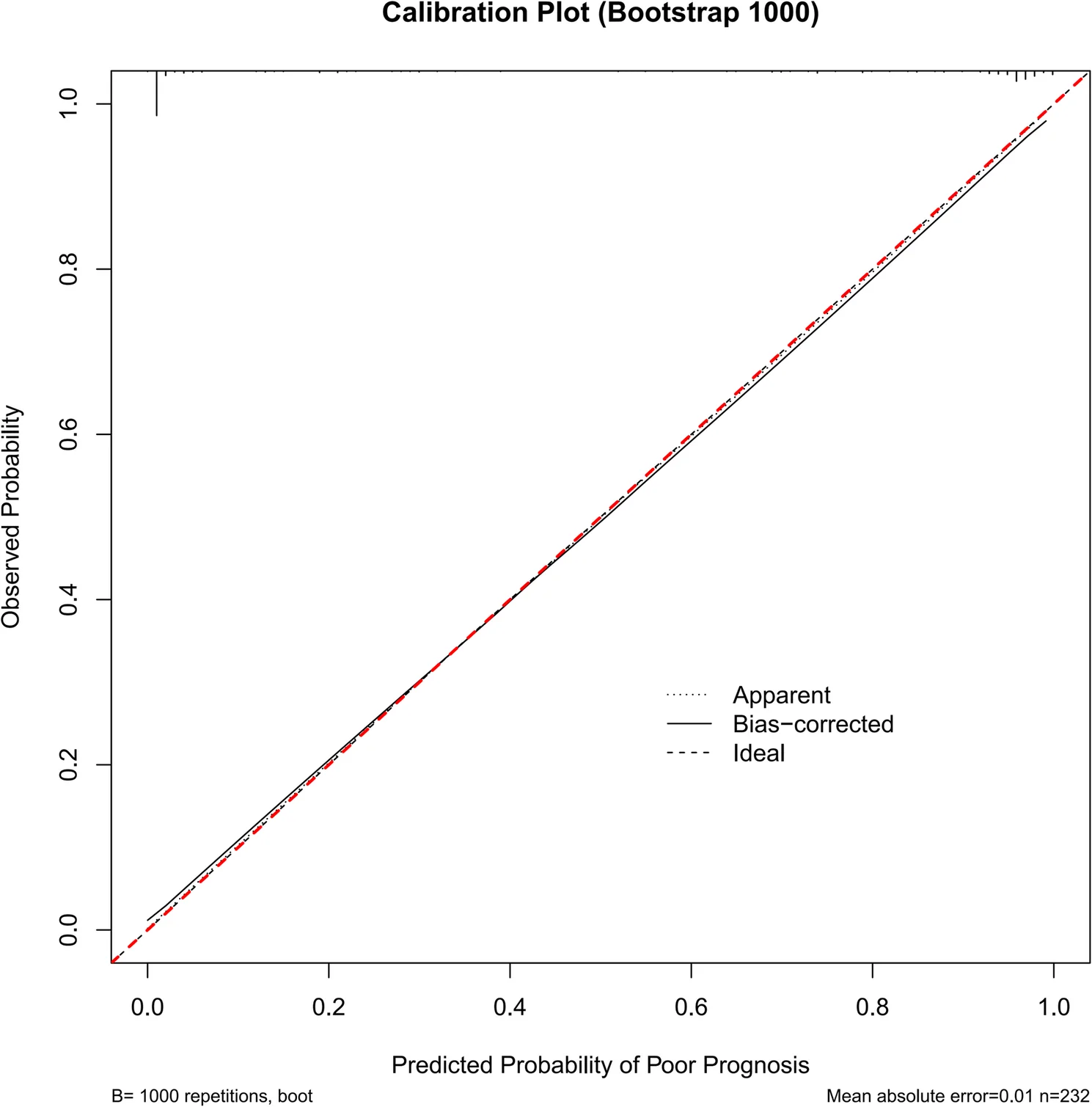
Calibration plot of the predictive model. The diagonal line represents perfect prediction. The mean absolute error (MAE) was 0.01

**Fig. 5:**
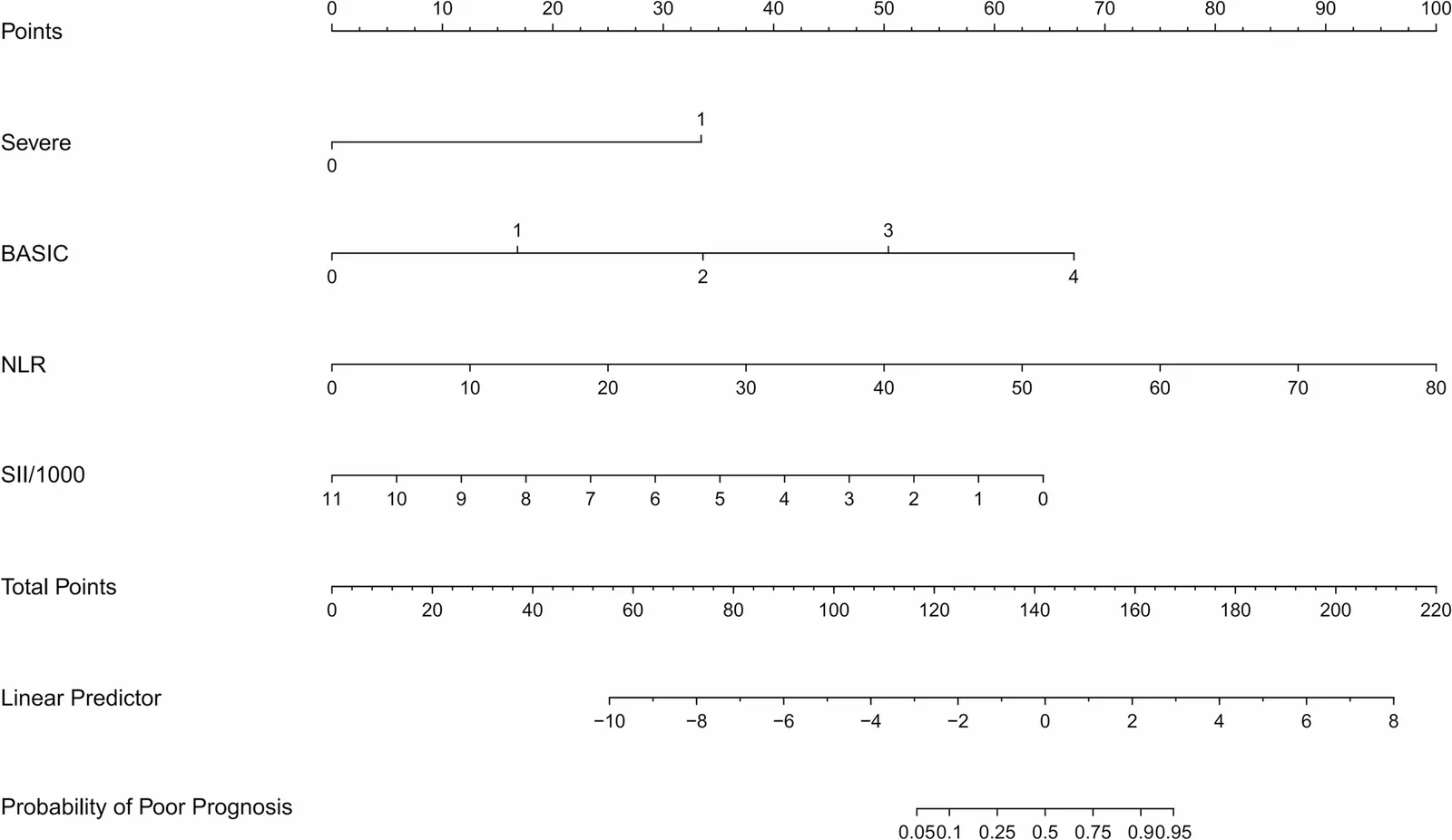
Nomogram for predicting the probability of poor prognosis at 1-year. To use the nomogram, locate the value of each predictor on its axis, draw a vertical line to the “Points” axis, sum the points, and determine the corresponding predicted probability. (Note: SII is presented as SII×10³. The axis is plotted in reverse order to directly reflect its regression coefficient in the multivariable model. Clinically, higher SII is associated with worse prognosis, and this reversed axis is a statistical artifact due to collinearity with NLR)

Bootstrap internal validation with 1000 replications confirmed the model’s robustness, with an optimism-corrected AUC of 0.976 (Fig. 4).The Hosmer-Lemeshow test showed a non-significant P-value of 0.486, indicating excellent calibration, which was further confirmed by the calibration curve (MAE = 0.01, Fig. 4). A nomogram incorporating the four independent predictors is shown in (Fig. 5). Based on the optimal Youden index threshold of 0.357, patients were stratified into high-risk and low-risk groups. The model achieved a sensitivity of 94.9%, specificity of 91.7%, positive predictive value of 89.5%, and negative predictive value of 96.1% (Table 6).

**Table 6:** Predictive performance of the nomogram-based risk stratification model for poor prognosis in cervical TSCI

Parameter	Value
Optimal cut-off probability (Youden index)	0.357
Sensitivity	94.9%
Specificity	91.7%
Positive predictive value (PPV)	89.5%
Negative predictive value (NPV)	96.1%

*Abbreviations*: *PPV* Positive predictive value, *NPV* Negative predictive value

## Discussion

This study investigated the relationship between MRI parameters, peripheral inflammatory biomarkers, and the severity and prognosis of cervical TSCI. Our findings demonstrated a considerable association between admission MRI variables, inflammatory response biomarkers, and injury severity. Furthermore, a robust correlation was identified between these factors and poor prognosis. Multivariate logistic regression analysis integrating all peripheral inflammatory biomarkers and MRI variables identified injury severity, BASIC score, NLR, and SII as independent predictors of 1-year prognosis in cervical TSCI. Based on these findings, a predictive model was developed by integrating these variables, significantly improving prognostic accuracy.

The AIS grade remains the primary clinical indicator for assessing cervical TSCI severity and neurological prognosis [3, 4, 24, 25]. Our study reaffirmed its strong association with long-term prognosis. However, its clinical utility has limitations. First, its accuracy may be compromised in intubated or sedated patients, potentially affecting physician assessment [5]. Second, TSCI patients may experience transient spinal shock symptoms, as some degree of spontaneous recovery from spinal shock can occur within hours [13]. Therefore, alongside improved diagnostic and predictive models for acute TSCI, identifying reliable biomarkers remains essential for enhancing assessment accuracy. Current advancements in alternative biomarkers, including admission blood tests, cerebrospinal fluid analysis, motor-evoked potential monitoring, and MRI, have shown promise in clinical applications [26–30].

MRI quantifies structural damage and is a key tool for evaluating TSCI severity and guiding treatment decisions. However, its predictive accuracy in assessing long-term neurological outcomes remains suboptimal. The roles of MSCC and MCC in neurological recovery remain debated, though prior studies suggest these parameters are crucial for estimating the likelihood of irreversible neurological dysfunction following cervical TSCI [31, 32]. However, Haefeli et al. [10] and Mabray et al. [33]assessed the prognostic value of various MRI indicators for early neurological outcomes in TSCI using nonlinear principal component analysis. Their findings suggested that MCC and MSCC were ineffective as prognostic indicators. Similarly, our study found that while MSCC strongly correlated with injury severity and follow-up AIS grades, neither MSCC nor MCC emerged as significant prognostic predictors in multivariate logistic regression analysis, aligning with our previous research. This observation may be because MSCC and MCC primarily reflect post-traumatic structural compression on MRI, potentially underestimating the actual extent of spinal cord compression during injury, which could lead to irreversible damage.

Talbott et al. [9] introduced the BASIC score in 2015 based on axial MRI findings, demonstrating its strong correlation with neurological symptoms at admission and discharge. A prior study indicated that each 10 mm increase in IMLL reduces the AIS grade conversion rate by 40% [34]. Moreover, IMLL has been identified as a key predictor of ambulatory recovery at discharge [35]. Our study revealed that BASIC score, sagittal grade, and IMLL negatively correlated with AIS grade at follow-up, with the BASIC score exhibiting the strongest correlation. The BASIC score was the only significant prognostic predictor in multivariate logistic regression. Although all three indicators primarily assess T2 high signals within the spinal cord, they differ in key aspects. The correlation between the BASIC score and sagittal grade differs in mild to moderate classifications; however, both incorporate hemorrhage and edema as critical scoring factors. Specifically, the BASIC score emphasizes the extent of preserved white matter, whereas the sagittal grade prioritizes the distinction between edema affecting a single vertebral level and that spanning multiple levels. The sagittal grade and IMLL are conceptually similar, with the sagittal grade accounting for hemorrhage. As expected, their combined use demonstrates convergent and construct validity, reinforcing their negative correlation with AIS grade at follow-up and confirming their predictive value. Ultimately, the BASIC score emerged as the most reliable imaging predictor of prognosis, as it was the only variable that remained significant in the multivariate model. This finding underscores its superiority over other MRI indicators in accurately capturing AIS grade changes at follow-up.

The inflammatory response triggered by TSCI frequently leads to secondary damage, characterized by immune cell infiltration and cytokine release, which can further exacerbate injury and induce pathological changes [36, 37]. Consequently, inflammation-related biomarkers may aid clinicians in predicting TSCI prognosis. Zhou et al. [12] reported significantly elevated WBC and neutrophil counts in patients with severe TSCI, suggesting a potential link between peripheral inflammatory biomarkers and neurological outcomes. Their study found acute neutrophilia correlated positively with TSCI severity but negatively with neurological recovery. Moreover, serum CRP was identified as a key inflammatory marker for predicting cervical TSCI prognosis [5, 38]. Emerging inflammatory biomarkers offer valuable insights into complex immune responses and are increasingly recognized as systemic inflammation markers due to their simplicity, reliability, and ease of calculation. Our multivariate logistic regression analysis identified NLR and SII as significant inflammatory predictors of TSCI prognosis, emphasizing their clinical relevance. Zhao et al. [39] demonstrated that patients with poorer outcomes had significantly higher NLR, making it an independent risk factor for unfavorable prognosis 6 months post-injury. Similarly, Liu et al. [6] found that NLR was a stronger predictor of AIS grade conversion. Zhou et al. [12] further reported that SII levels were substantially elevated in patients with lower admission AIS grades, while Wang et al. [40] observed that elevated SII levels were independently associated with a reduced likelihood of AIS grade improvement after TSCI. Although NLR and SII remained significant in the multivariable analysis, their individual ROC values were relatively low, indicating limited standalone discriminatory ability. These inflammatory biomarkers may therefore be more informative when interpreted in combination with admission injury severity and MRI-based structural indicators rather than as independent clinical prediction tools.Our study confirmed that the integration of MRI-based BASIC scores, NLR, and SII is a reliable prognostic tool. Furthermore, our analysis demonstrated that a predictive model incorporating injury severity, BASIC score, NLR, and SII achieved a higher AUC, demonstrating superior prognostic accuracy. However, multiple factors may influence the validity of AUC outcomes, including the timing [41] and type [42] of decompression surgery. These factors, affected by spinal cord signaling abnormalities and the extent of compression, could ultimately impact patient prognosis. In this study, all peripheral blood samples were collected within 24 h after injury and prior to surgery, indicating that the inflammatory biomarkers reflect the early preoperative systemic inflammatory response. Although patients with severe multiple injuries, which hindered reliable AIS assessment, were excluded, minor concomitant injuries may still have influenced peripheral inflammatory biomarker levels.

### Limitation

First, although 232 patients were retrospectively analyzed, it was a single-center study with a relatively small sample size. It lacked prospective validation, potentially introducing selection and information biases, thereby limiting the generalizability of the findings.Second, Although bootstrap validation confirmed the internal stability of the model, with a corrected AUC of 0.976, the corrected calibration slope of 0.855 and the single-center design suggest that some degree of optimism may remain in the current estimates. Therefore, the proposed model should be considered exploratory, and external validation in independent, multicenter cohorts is required before its clinical application. Notably, the coefficient of SII reversed in the multivariate model, likely due to collinearity with NLR, as both derive from the same cell counts. When highly collinear, suppression may reverse the sign. Thus, while elevated SII alone is a risk indicator, its independent role here should be interpreted cautiously. BASIC and NLR carry the main predictive weight, with SII serving only as a minor modifier.In addition, although all patients underwent surgical treatment, detailed treatment-related variables were not consistently available because of the retrospective nature of the study. Specifically, standardized information on the timing and duration of decompression surgery, surgical approach, perioperative mean arterial pressure targets and maintenance duration, vasopressor use, and corticosteroid administration was unavailable for a proportion of patients. Consequently, these potentially important treatment-related confounders could not be reliably incorporated into the multivariable analysis, although they may influence neurological recovery. Finally, the AIS grading system is relatively broad and subjective, which may weaken the observed correlations. Follow-up AIS grades were obtained through outpatient visits or structured telephone follow-up, and telephone-based assessments may be less precise than direct standardized neurological examinations. Although patients with severe concomitant injuries affecting AIS evaluation were excluded, minor injuries may still have influenced systemic inflammatory biomarkers. In addition, BMI could not be included because height and weight were inconsistently recorded in the retrospective medical records. Future prospective, multicenter studies using standardized surgical and perioperative protocols, serial biomarker measurements, AIS grade conversion, and detailed imaging and functional assessments are needed to validate and refine the model and improve its clinical applicability.

## Conclusion

The combination of preoperative BASIC score, NLR, SII, and injury severity demonstrated promising value for prognostic stratification in patients with spinal cord injury. Bootstrap internal validation supported the stability and discriminative performance of the model. These findings suggest that the model may provide a useful tool for early risk assessment and individualized clinical decision-making. Moreover, this is the first study to compare or integrate the BASIC score, SII, and acute TSCI severity. The ROC analysis demonstrated that combining the variables of BASIC score, NLR, SII, and severity of injury enhances predictive accuracy for neurological recovery post-TSCI.The present findings support the potential prognostic value of combining preoperative MRI parameters and inflammatory biomarkers. However, the proposed model should be regarded as an exploratory preoperative prognostic model. Prospective multicenter studies incorporating standardized surgical and perioperative treatment variables are required before clinical application.Future prospective, large-scale, multi-center studies are warranted to refine these prognostic markers and validate our results.

## Supplementary Information

Supplementary Material 1.

Supplementary Material 2.

## Data Availability

The datasets used and/or analysed during the current study available from the corresponding author on reasonable request.

## Abbreviations

AIS: American spinal injury association Impairment Scale
AUC: Area under the curve
BASIC: Brain and spinal cord injury center
CRP: C-reactive protein
GCS: Glasgow Coma Scale
IMLL: Intramedullary lesion length
ROC: Receiver operating characteristic
MCC: Maximum canal compromise
MRI: Magnetic resonance imaging
MSCC: Maximum spinal cord compression
SCIM: Spinal cord independence measurement
TSCI: Traumatic spinal cord injury
WBC: White blood cell
NASCIS: The National Acute Spinal Cord Injury Study
NLR: Neutrophil-to-lymphocyte ratio
PLR: Platelet-to-lymphocyte ratio
LMR: Lymphocyte-to-monocyte ratio
SII: Systemic immune inflammatory index
GCS: Glasgow Coma Scale
ISNCSCI: International Standards for Neurological Classification of Spinal Cord Injury
ANOVA: Analysis of variance
IQR: Interquartile range
SD: Standard deviation
