# Supplementary material for: Combined predictive value of MRI parameters and inflammatory biomarkers in assessing the severity and prognosis of acute cervical traumatic
Source: BMC Musculoskelet Disord. 2026 Aug 14;27:977. doi: 10.1186/s12891-026-10362-6 (PMC13756934; doi:10.1186/s12891-026-10362-6)
Supplement: Supplementary file 1 [file 12891_2026_10362_MOESM1_ESM.pdf]

Supplementary File 1

Hosmer-Lemeshow Calibration Test

Calibration was assessed using the Hosmer-Lemeshow goodness-of-fit test.

Table 1. Hosmer-Lemeshow calibration test results.

Sum of squared errors	Expected value under H0	SD	Z	P value
12.7869887	12.3905234	0.5694464	0.6962292	0.4862853
