# Supplementary material for: Combined predictive value of MRI parameters and inflammatory biomarkers in assessing the severity and prognosis of acute cervical traumatic
Source: BMC Musculoskelet Disord. 2026 Aug 14;27:977. doi: 10.1186/s12891-026-10362-6 (PMC13756934; doi:10.1186/s12891-026-10362-6)
Supplement: Supplementary file 2 [file 12891_2026_10362_MOESM2_ESM.pdf]

Supplementary File 2

Bootstrap Internal Validation

Internal validation was performed using 1,000 bootstrap resamples. The original, training, test, optimism, and optimism-corrected estimates are presented below.

Table 1. Bootstrap internal validation results (1,000 resamples).

Index	Original	Training	Test	Optimism	Optimism-corrected	n
**Dxy**	0.9582	0.9585	0.9513	0.0072	0.9511	1000
**R2**	0.8475	0.8561	0.8334	0.0227	0.8248	1000
**Intercept**	0.0000	0.0000	0.1182	-0.1182	0.1182	1000
**Slope**	1.0000	1.0000	0.8549	0.1451	0.8549	1000
**Emax**	0.0000	0.0000	0.0561	0.0561	0.0561	1000
**D**	0.9927	1.0095	0.9653	0.0442	0.9485	1000
**U**	-0.0086	-0.0086	0.0206	-0.0292	0.0206	1000
**Q**	1.0013	1.0181	0.9447	0.0734	0.9279	1000
**B**	0.0551	0.0518	0.0581	-0.0064	0.0615	1000
**g**	5.3893	6.6956	5.1828	1.5128	3.8765	1000
**gp**	0.4680	0.4672	0.4627	0.0045	0.4635	1000
